# Digital Traps: The Critical Role of Online Encounters in the Entrapment of Minors in Sex Trafficking

**DOI:** 10.1177/15248380241290245

**Published:** 2024-10-15

**Authors:** Kyla Baird, Jennifer Connolly

**Affiliations:** 1 Department of Psychology, York University, Toronto, ON, Canada

**Keywords:** human trafficking, commercial sexual exploitation/human trafficking, adverse childhood experiences, child maltreatment

## Abstract

We are grateful to the Editors of TVA for the opportunity to respond to the commentary on our systematic review of the sex trafficking of minors, specifically regarding the initial recruitment location. Upon revisiting the 7 out of 23 reviewed studies that address recruitment locations, we find that the discrepancy with the commentators’ views stems from differing interpretations of the term “initial.” We affirm that these seven studies, which include the internet as a prominent initial recruitment site, are valid and appropriate for inclusion. We also emphasize that, irrespective of recruitment location, we and the commentators share deep concerns about the severe impact of sex trafficking on minors, recognizing it as a heinous crime against vulnerable populations. Traffickers use both online and in-person methods to manipulate and exploit youth. Our review highlights the internet as a primary platform for traffickers to form relationships with minors, comparable in danger to in-person interactions. The 23 reviewed papers focus on documenting these predatory relationships and the critical role of supportive, healing relationships in prevention and intervention.

We wish to extend our thanks to the Editors of TVA for their invitation to respond to the commentary on the conclusions we draw in our systematic review of sex trafficking of minors regarding the initial location of recruitment (Baird & Connolly, 2023) We have carefully considered their comments and have re-examined the studies in our systematic review that address this point (7 out of 23 papers). We conclude that the discrepancy between our review and those of the commentators lies in the different ways that the word “initial” is being used. We are confident that the seven studies that indexed location are both valid and appropriate for inclusion in our systematic review. Moreover, we continue to support the conclusions of the reviewed papers that the internet is a primary location for predatory action in the initial phase of recruitment and that the relationships that traffickers form with youth online are as dangerous as those they might form through in-person interactions. We outline in more detail our responses to these issues below. Prior to doing so, we would like to thank the commentators for describing our systematic review as “excellent.” We appreciate your endorsement of our work. Second, we would like to highlight that apart from the question of recruitment location, we and the commentators are perfectly aligned in our deep concerns for the consequences of sex trafficking on our children and youth, unquestionably a most heinous crime committed against the most vulnerable members of our society. Like the commentators, we are highly invested in prevention and intervention to reduce the risk of trafficking (Benvenuto & Connolly, 2024) and in providing healing and recovery to youth who have been trafficked (McDonald et al., 2024). Finally, we acknowledge that the available research on the sex trafficking of minors consistently highlights how deeply trafficking is rooted in deceptive relationships. Traffickers pose as supporters or benefactors, using manipulation to recruit, entrap, and ultimately enmesh them in exploitation. A reading of our systematic review makes evident that the 23 papers that we included are primarily focused on documenting the predatory relationships traffickers establish with their intended victims, the vulnerability of youth to these manipulations, and the crucial role that healing relationships play in prevention and intervention efforts.

Turning to our points of disagreement, they center on the location of contact between the youth and the trafficker and the implications for prevention programs. In surveying the 23 articles identified for inclusion in the systematic review, seven of them (Baird et al., 2020; Moore et al., 2020; O’Brien, 2018; O’Brien & Li, 2020; Rosenblatt, 2014; Tidball et al.,2016; Wells et al., 2012) refer to the location of contact between the trafficker and the youth. As noted in Table 1 of our systematic review (Baird & Connolly,2023) locations of contact included bus stops, schools, malls, and the Internet. Our statement that the most frequent location was the internet is simply a statement of the fact that all of the studies mentioned online contact, whereas other locations were not universally mentioned. Our reference to online contact as “initial” is in the context of discussing the preliminary phase of the recruitment of the youth, as compared to later phases of entrapment and enmeshment. During this initial phase of contact, the trafficker creates a relationship with the intended victim and following this, increases the pressure through various means (see Table 1 in our systematic review) to force them into sexual acts. By contrast, the authors of the commentary have a different lens which they bring to these seven studies. They raise the question of whether the very first location of contact is online. This question arises from the work of Finkelhor et al. (2021), who suggest that the very first point of contact is in-person, whether or not it would then be followed by online contact. This is an interesting distinction and worthy of further consideration. As the commentators note, it is not one that can be answered with the extant literature. This is because the reviewed papers, by and large, do not distinguish between the very first contact and the series of contacts in the initial recruitment phase. The three studies which do make this distinction indicate that the first point of contact was online but they are discounted by the commentators as they are deemed to have methodological limitations. We counter this assertion in the following paragraph.

The 7 studies of the 23 in our review that mention location employ quantitative (2), qualitative (4), or mixed-methods (1) research designs. The studies utilizing quantitative data employ secondary information provided by chart reviews of law enforcement or medical records. The commentators view them of lesser value since they do not interview the victims directly. In our opinion, secondary data analysis provides very useful information drawn from large samples of cases from which the same information can be extracted for each case, thus increasing the confidence we have in the data findings. We also note that ethical and legal considerations create important barriers to interviewing survivors of trafficking out of concern of re-traumatization or placing them at legal risk if their trafficker is facing criminal prosecution (Zimmerman & Watts, 2003). The qualitative studies, as the commentators note, are valuable because they provide firsthand accounts from trafficking survivors, but in our view, have their limitations. They have very small samples and are largely retrospective accounts from adults who were trafficked as youth and who may range in age from 20s to 60s. These studies are important because they speak to the lived experiences of trafficked minors and shine a light on the needs and vulnerabilities that made them susceptible to the false promises of traffickers. At the same time, the qualitative method does not yield data that can be generalized to the population of youth at large (Morse, 2015). Considering the small scope of the available studies, we argue that dismissing the secondary data analyses or the qualitative studies arbitrarily reduces the information on location-based insights. Finally, the commentators state that our review included studies of questionable quality. Restricting the inclusion criteria in a systematic review to those studies that meet a quality benchmark is typically done when the topic of the review has a very substantial body of research and the review can focus on “gold standard” research, for example, randomized control trials. Exploratory reviews such as ours provide a starting point for better understanding the recruitment and entrapment of minors into sex trafficking. We argue that the importance of better understanding these processes is of high societal importance and we should proceed with all available information, hoping of course, that future studies build on current ones and can employ more robust research designs.

A final point of disagreement rests in the commentators’ assertion that articles that draw attention to the dangers of online contact with strangers lead adults, in their role of supporting youth, to disregard alerting them to the dangers posed by in-person contact with individuals who are intending to lure the youth into sex trafficking. Their view is that there is a lack of evidence that the very first contact between a trafficker and youth is online and so prevention programs should continue to emphasize the dangers of forming relationships with individuals met in person. They further suggest that highlighting the dangers of social media is likely to induce a “moral panic” among Americans such that online activity is vilified, and any potential benefits of virtual contacts and relationships are discounted. We dispute both points. First, at no point in our review, do we diminish the role of an in-person relationship between the trafficker and the youth in the recruitment process. Indeed, building on the conclusions of the 23 papers in our systematic review, we emphasize the centrality of the manipulative relationships traffickers offer, luring youth with promises of financial and emotional rewards. Warning youth about the dangers of meeting strangers in person is of limited value and youth need to be aware of the many hazardous environments in which hazardous relationships can be formed. Turning to the second point, research and practice now highlight the significant harm to youth that can accrue due to social media exposure. In June 2024, the US Surgeon General issued a public health advisory on social media and youth mental health (U.S. Department of Health and Human Services, 2024). The advisory draws attention to the negative effects of extensive social media exposure on mental health, including anxiety, body dysmorphia, and depression. In addition, the advisory warns of the dangers of stranger contact on the internet, stating that “social media platforms can be sites for predatory behaviors and interactions with malicious actors who target children and adolescents (e.g., adults seeking to sexually exploit children, to financially extort them . . .)” (p. 9). Surgeon General Advisories are issued rarely and only when there is a substantial public health risk that requires concerted preventative action. Youth spend significant amounts of time on the internet accessing social media. The dangers are real. It is the foremost responsibility of all adults who interact with young people to alert them to the risks of online exploitation and help them recognize warning signs.

To summarize, we appreciate the distinction that the commentators are making between the very first contact that a trafficker makes with youth and the further contact that occurs in the initial phases of luring and recruitment. We agree that there is simply insufficient evidence in the extant literature to shine a light on this distinction and suggest this might be a fruitful topic for future research. We remain confident in our summary of the extant research that the Internet is pervasive in the initial phases of luring and recruitment. We support all education and prevention efforts that help youth identify unsafe relationships, whether they be in person or online. Despite the methodological challenges, we encourage researchers to continue their efforts to understand and eradicate all forms of human trafficking. We encourage researchers to develop evidence-based education programs that will prevent abuse and exploitation of youth, and interventions that will correct the harms that have occurred to trafficked youth and allow them to live their lives free of abuse and exploitation.
